# Performance Consistency of International Soccer Teams in Euro 2012: a Time Series Analysis

**DOI:** 10.2478/hukin-2013-0061

**Published:** 2013-10-08

**Authors:** Mohsen Shafizadeh, Marc Taylor, Carlos Lago Peñas

**Affiliations:** 1 Academy of Sport and Physical Activity, Faculty of Health and Wellbeing, Sheffield Hallam University, Collegiate Campus, Sheffield, UK.; 2 Academy of Sport and Physical Activity, Faculty of Health and Wellbeing, Sheffield Hallam University, Collegiate Campus, Sheffield, UK.; 3 Faculty of Educational Sciences and Sports, University of Vigo, Spain.

**Keywords:** consistency of play, match result, performance indicators, soccer

## Abstract

The purpose of this study was to examine the consistency of performance in successive matches for international soccer teams from Europe which qualified for the quarter final stage of EURO 2012 in Poland and Ukraine. The eight teams that reached the quarter final stage and beyond were the sample teams for this time series analysis. The autocorrelation and cross-correlation functions were used to analyze the consistency of play and its association with the result of match in sixteen performance indicators of each team. The results of autocorrelation function showed that based on the number of consistent performance indicators, Spain and Italy demonstrated more consistency in successive matches in relation to other teams. This appears intuitive given that Spain played Italy in the final. However, it is arguable that other teams played at a higher performance levels at various parts of the competition, as opposed to performing consistently throughout the tournament. The results of the cross-correlation analysis showed that in relation to goal-related indicators, these had higher associations with the match results of Spain and France. In relation to the offensive-related indicators, France, England, Portugal, Greece, Czech Republic and Spain showed a positive correlation with the match result. In relation to the defensive-related indicators, France, England, Greece and Portugal showed a positive correlation with match results. In conclusion, in an international soccer tournament, the successful teams displayed a greater degree of performance consistency across all indicators in comparison to their competitors who occasionally would show higher levels of performance in individual games, yet not consistently across the overall tournament. The authors therefore conclude that performance consistency is more significant in international tournament soccer, versus occasionally excelling in some metrics and indicators in particular games.

## Introduction

In the last decade the application of notational analysis to understand the different aspects of performance in individual or team sports has become more popular among sport and exercise scientists. Many analysts who worked at various levels of sport performance (i.e. grassroots/amateur through to elite sport) have used it for different purposes including technical and tactical evaluation, movement analysis, feedback provision, norm development and modeling (Hughes and Bartlett, 2008). Currently many sports and specifically many soccer clubs and national teams use different technologies to explore the tactical features of a game in order to enhance the likely group performance in future matches, by understanding their own or their opponents' strengths and weaknesses (Carling et al., 2005). The need for match analysis is important, especially as this is often used as a powerful communication and feedback tool by many coaches to instruct or educate players during practice sessions to analyze the quality of performance during or after the match. Its capability as a feedback or education tool within the coaching structure is due to the type and quality of feedback, providing relevant quantitative and qualitative data, with visual and video feedback (Liebermann and Franks, 2008). This type of feedback is usually easily understood by multiple stakeholders (e.g. players, coaches, administrators, owners etc.) Identification of key indicators is a common way to assess the performance in sport. These are characterized as single or combination of action variables that are related to successful outcomes as the forms of scoring and playing perspectives in notational analysis (Hughes and Bartlett, 2002).

Different studies have been carried out in various soccer structures. Some of these studies have focused on international tournament soccer competitions, some on top level cross broader tournament competitions (e.g. UEFA Champions League) and some on top level domestic league soccer. Consequently a number of factors is considered and proposed as being relevant for successful performance (Lago-Penas et al., 2010; Erkmen, 2009; Kannekens et al., 2011; Lago-Ballesteros and Lago-Penas, 2010; Tenga et al., 2009; Rampinini et al., 2009; Lago-Penas et al., 2011; Rampinini et al., 2007). Lago-Ballesteros and Lago-Penas (2010) in La Liga, Spain, for all clubs in season 2008–2009 found that top level teams had better performance in goal scoring, total shots, shots on target, possession and assists relative to middle and lower ranked teams. Lago-Penas et al. (2011) studied group stage teams in UEFA Champions league between 2007 and 2010 in terms of winning, drawing and losing, rather than final ranking. Their results showed that the best discriminative indicators were shots on target, the number of crosses and ball possession.

Although numerous studies show similar findings in relation to successful performance indicators in soccer, many of them also suggest that a number of differences that occur in different leagues, due to the local context, culture and tactics deployed. For example Dellal et al. (2011) found a different playing pattern between England Premier league and Spain La Liga in terms of physical and technical factors. The results revealed that Premier league players covered greater distances in sprinting; in contrast La Liga players covered more total distance during ball possession. They had the same amount of successful passes, while La Liga players won more aerial heading duels.

In addition to match analysis in domestic soccer leagues, some scholars studied the key performance indicators in arguably bigger events and in national soccer teams in continental tournaments or world cups. Selecting these kinds of tournament as a context to find successful performance indicators is very different from domestic leagues in terms of the number of matches, the quality of opponents, the physical demands of match and time limit (Hughes and Franks, 2005; Luhtanen et al., 2001; Armatas et al., 2007; Scoulding et al., 2002; Castellano et al., 2012; Shafizadeh et al., 2012; Hook and Hughes, 2001; Stanhope, 2001). Analysis of soccer performance in different world cups (Hughes and Franks, 2005; Castellano et al., 2012) and Euro 2000 (Hook and Hughes, 2001) showed that converting possessions into shots on goal, longer possessions, total shots and shots on target were the best discriminative factors between successful and unsuccessful teams in tournaments.

Previous studies have used different ways in order to find the key performance indicators for success in soccer. The common design for data analysis was primarily focused on descriptive and comparative statistics such as frequency, percentage, means comparison, regression models and discriminative analysis. The design of the study mainly consisted of the average or sum of performance indicators which were compared or correlated between different conditions such as successful/unsuccessful and win/draw/loss (Hook and Hughes, 2001; Hughes and Franks, 2005; Castellano et al., 2012; Lago-Ballesteros and Lago-Penas, 2010; Lago-Penas et al., 2010). These kinds of studies played an important role in exploring the best performance indicators for success in soccer, therefore, they also helped present different variables necessary to understand successful performance from match analysis techniques as opposed to different points of view and with other methods of data analysis. Luhtanen et al.’s (2001) method was based on an analytical study of EURO 2000 in order to find strengths and weaknesses of all teams in different performance indicators. For example, France who won the tournament was the most successful team in relation to metrics associated with successful pass completion (made and received), as well as running with the ball and tackling. Italy was stronger in their defensive attributes, particularly intercepting the ball from the opposition passes and also tackling. The Netherlands showed their overall best metrics to be in the categories of ball retention (possession), passing and shooting. Germany was also strong in relation to ball retention (possession), passing and goal scoring (from shots). What this analysis shows is a description of performance; it does not however suggest which attributes will lead to overall success in international tournament soccer. The challenge with this analysis is that this does not take into account the opposition, the deployment of their tactics or the dynamic ‘flow’ of the game and the opposition’s tactics.

Time series analysis is a method that has been used extensively for motion analysis in biomechanics of sport (Stergiou, 2004). This method is based on analysis of successive attempts in a specific period of time that represents the persistency or change in the series of data due to internal or external factors and through different methods of analysis such as autocorrelation and cross-correlation. In spite of applications of this method in human movement analysis little is known about its appropriateness in match analysis. Yue et al. (2008) used time series as a mathematical method to analyze individual and collective behaviors to explore the possession, speed and covered distance in a certain period of time in soccer.

Because of the complexity of soccer and the effects of situational parameters such as match location, quality of opposition and match status on the performance (Taylor et al., 2008; Lago-Penas et al., 2010), the necessity to understand the game pattern of top level soccer teams could help to find the key indicators for persistent performance. According to FIFA ranking, Spain has been the first ranked team in Men's soccer between all teams in Europe and in the world for several successive years and won three important tournaments including EURO 2008 and 2012 as well as World Cup 2010. However, there is some report about the consistency of Spain from 2006 to 2012 in big tournaments (Prozone, 2012) due to the pattern of play such as possession play, using space inside the box, cross and improving defensive skills. Yet, it is valuable to understand the consistency of performance of Spain and other teams who achieved success in part in the last couple of decades in EURO tournaments and through other methods such as a time series analysis rather than a descriptive comparison. This study aimed to answer the following questions: is consistency a discriminative factor for international tournament soccer teams and whether it determines the match result.

## Material and Methods

### Participants

Eight national soccer teams in the 2012 EURO soccer tournament in Poland and Ukraine were selected for this study. The teams included Spain, Italy, Germany, England, Greece, Portugal, France and the Czech Republic as these teams all reached the quarter final stage. All matches of each team were recorded from live broadcasting on BBC1 and ITV1. A total of 38 matches from preliminary to final stages were selected for analysis.

### Measures

The observation and analysis were taken from recordings of the soccer matches using the Sports Performer Software (Premier Concepts Pty Ltd, Australia). This software can record the frequency of movements on the basis of defined criteria. This software permits the collection and immediate analysis of data gathered from the observation of soccer matches either live or from DVD recordings. The computer keyboard was configured to permit the recording of multiple and overlapping frequency behaviors through pressing the appropriate keys.

### Procedures

Sixteen key performance indicators for analysis included three different categories of soccer performance. Goal-related indicators included total shots, shots on target and shot accuracy. Offensive-related indicators included ball possession, total number of passes, pass accuracy, long passes, crosses, cross accuracy and corners. Defensive-related indicators included tackles, tackles won, interceptions, clearances, duels won and aerial duels won.

### Statistical Analysis

The occurrences of all indicators were analyzed through absolute and relative frequencies. In the time series analysis, the forms of autocorrelation and cross-correlation were used to compute the consistency or persistency of performance in each team.

Autocorrelation is a statistical method to compute the relationship between a series of observations in a row with one, two and more time intervals, which is known as a lag. For the purpose of this study only the autocorrelation lag 1 was analyzed for the association between matches 1 and 2, 2 and 3, 3 and 4, 4 and 5, 5 and 6. A positive correlation was considered as ‘persistence of performance’ in successive matches. The higher values indicated a strong association or greater persistency in a specific performance indicator in successive matches.

Cross-correlation was used to calculate the relationship between performance indicators and the result of each match (win=3, draw=2, lose=1) in the lag 0. Higher correlation is considered as strong prediction of the game result in successive matches. SPSS software (V.18, IBM) was used to analyze all the data.

## Results

**Figure 1:**
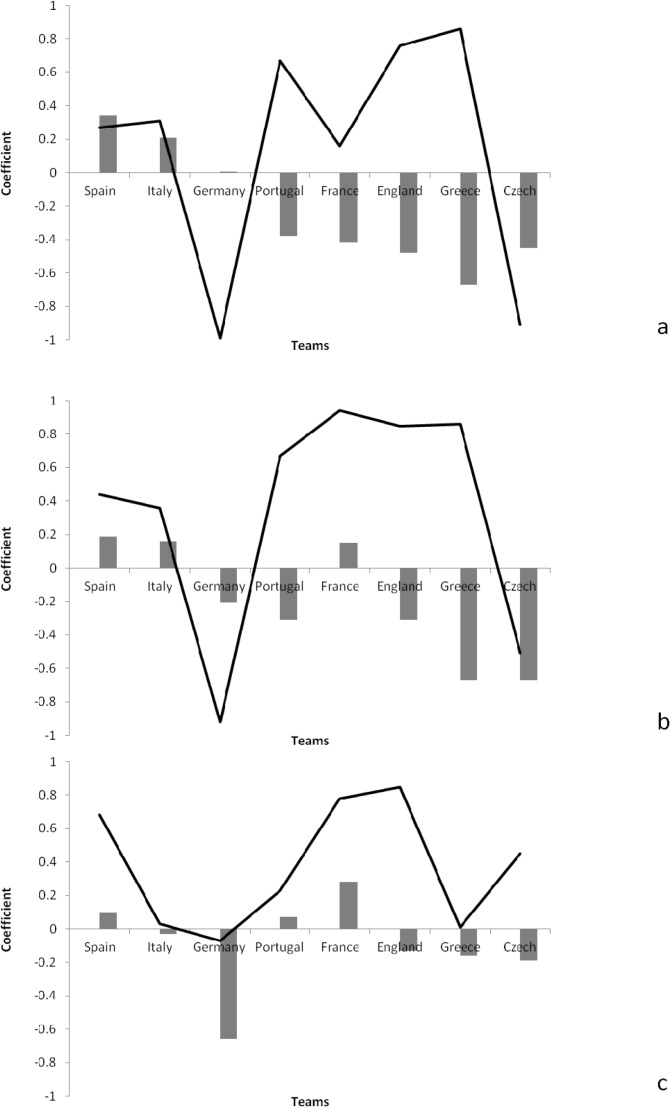
Autocorrelation functions (bar) and cross-correlation functions (line) on goal-related indicators in different teams; (a) shot, (b) shot on target, (c) shot accuracy

**Figure 2:**
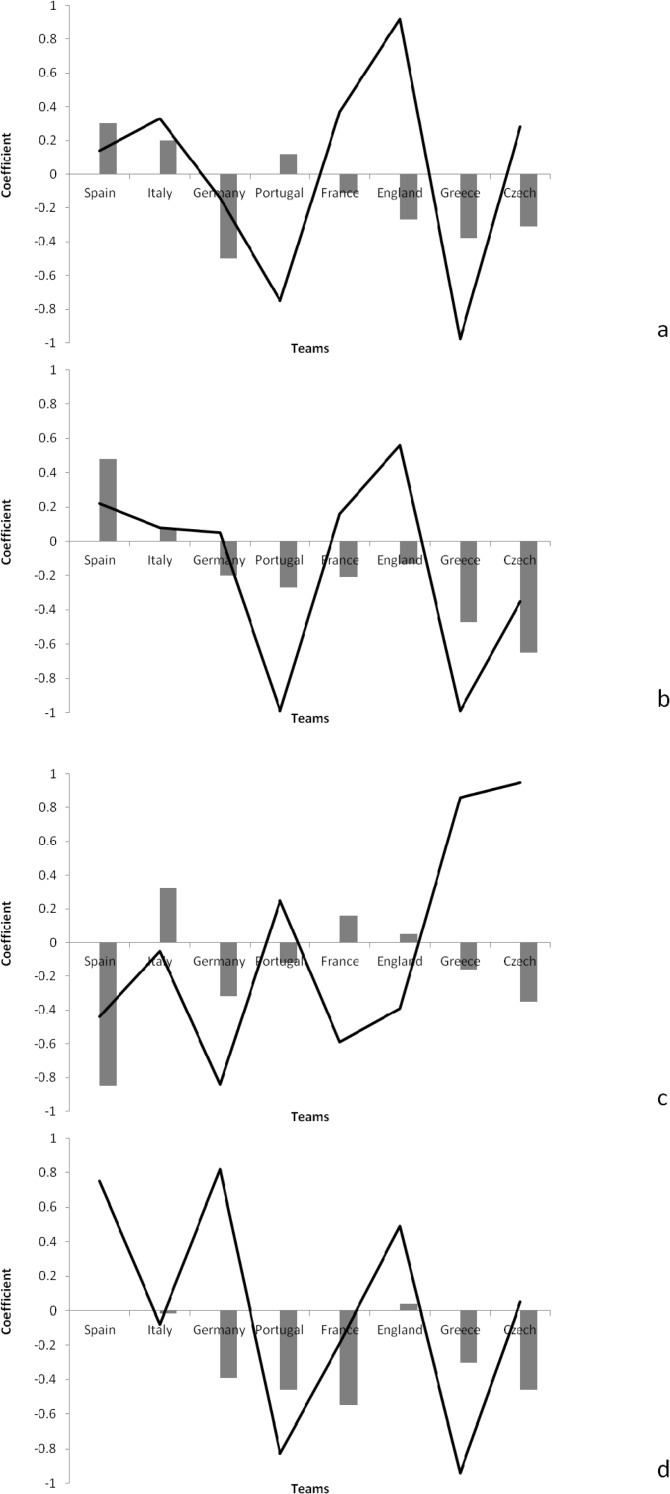

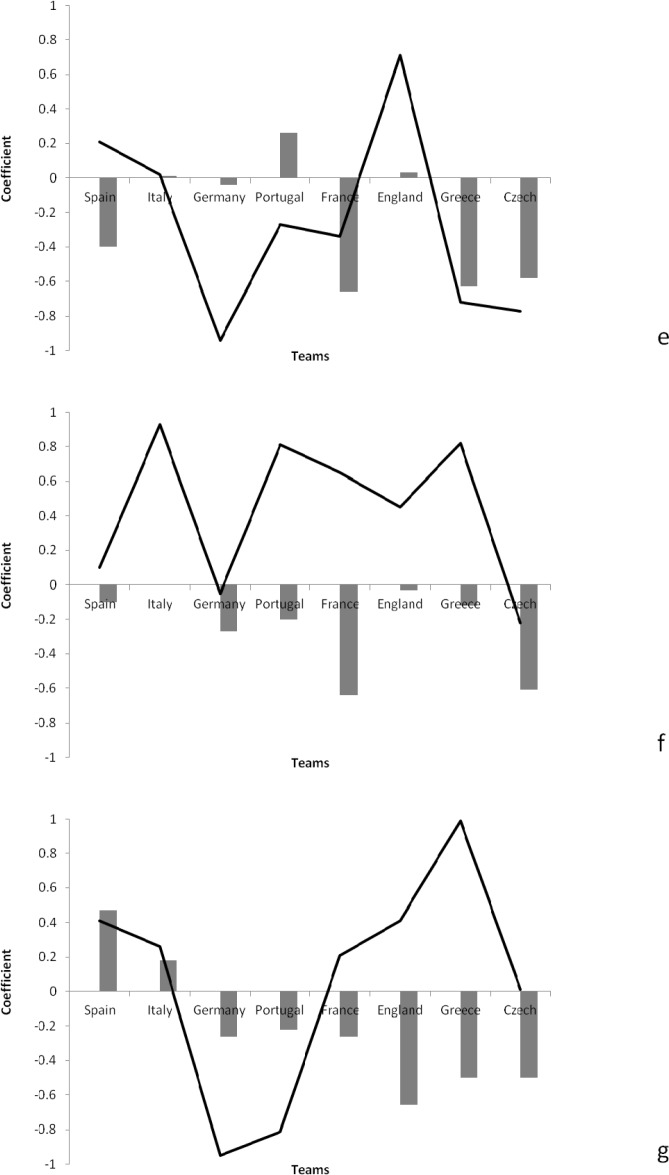
Autocorrelation functions (bar) and cross-correlation functions (line) on offensive-related indicators in different teams; (a) possession, (b) pass, (c) long pass, (d) pass accuracy, (e) cross, (f) cross accuracy, (g) corner

**Figure 3:**
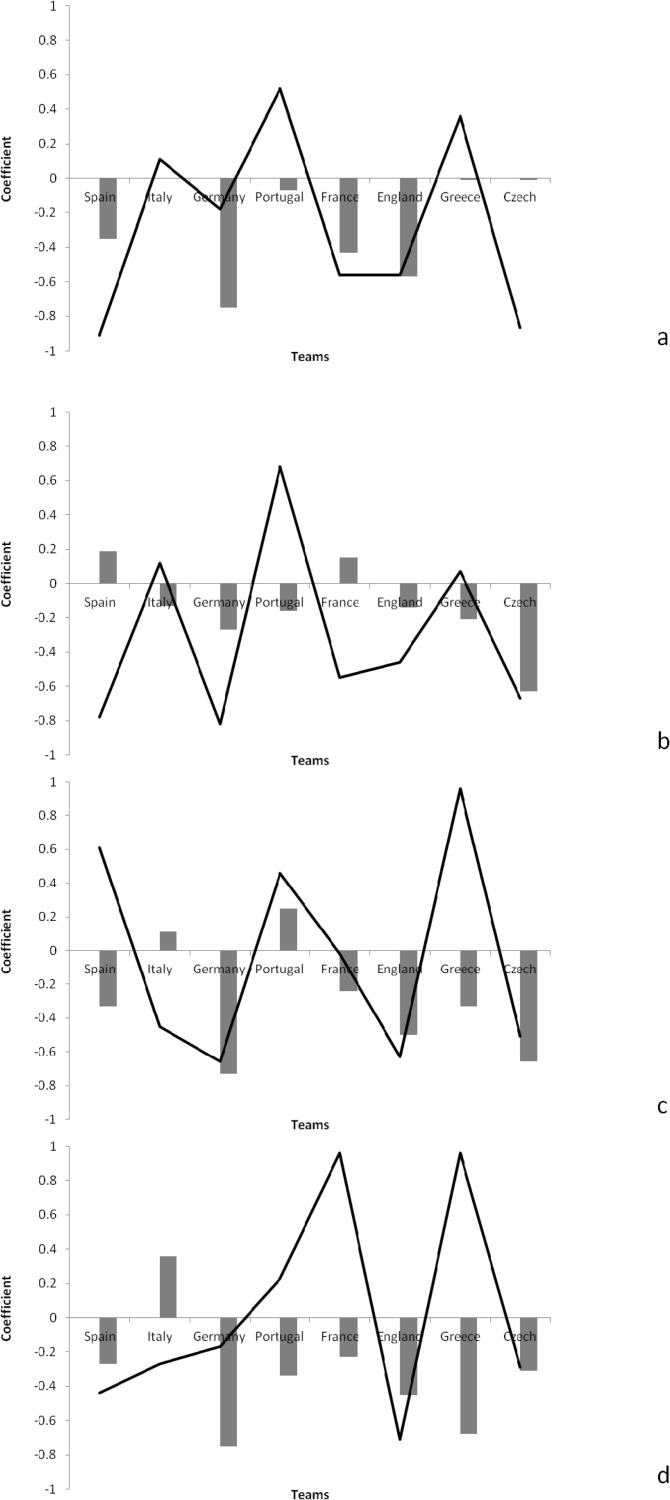

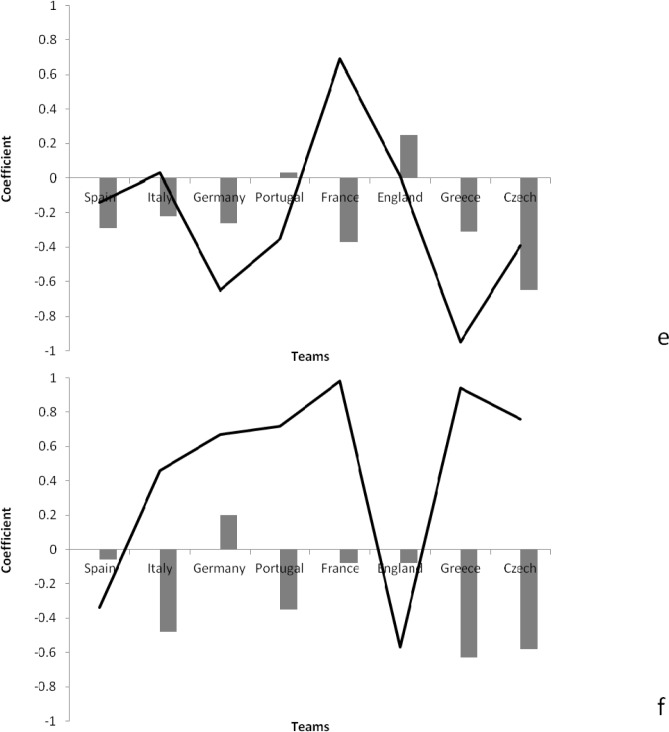
Autocorrelation functions (bar) and cross-correlation functions (line) on defensive-related indicators in different teams; (a) duel won, (b) aerial duel won, (c) interception, (d) tackle, (e) tackle won, (f) clearance

The results of different performance indicators are presented in Table 1 and Figures 1 to 3. Table 1 shows the mean and standard deviation of performance indicators in all teams. In average matches, Spain was in the first and second rank in relation to shots on target, shot accuracy, duels won, aerial duels won, possession, corners, total passes, pass accuracy and tackles won. Italy was in the first and second ranks in relation to shots, shots on target, interceptions and cross accuracy. Germany was in the first and second ranks in relation to ball possession, duels won, aerial duels won, corners, total passes and crosses. Portugal was in the first and second ranks in relation to long passes and crosses. France was in the first and second ranks in relation to shot, shot accuracy and passing accuracy. England was in the first and second ranks in relation to cross accuracy, tackles and clearances. Greece was in the first and second ranks in interceptions, long passes, tackles won and clearances. The Czech Republic was not in the first nor second rank in any of the performance indicators.

Figure 1 shows the autocorrelation and cross-correlation functions in relation to goal-related indicators for the different teams.

There were positive autocorrelations in relation to shots for Spain (ACF=0.34) and Italy (ACF=0.21). The results of cross-correlation also showed there were positive correlations in relation to shots and the result for Greece (CCF=0.86), England (CCF=0.76), Portugal (CCF=0.67), Italy (CCF=0.31) and Spain (CCF=0.27).

There were positive autocorrelations in relation to shot accuracy for France (ACF=0.28), Spain (ACF=0.10) and Portugal (ACF=0.07). The results of cross-correlation also showed there were positive correlations in relation to shot accuracy and the result for England (CCF=0.85), France (CCF=0.78), Spain (CCF=0.68), Czech (CCF=0.45) and Portugal (CCF=0.23).

Figure 2 shows the autocorrelation and cross-correlation functions for offensive-related indicators for the different teams.

There were positive autocorrelations in relation to ball possession for Spain (ACF=0.30), Italy (ACF=0.20) and Portugal (ACF=0.12). The results of cross-correlation also showed that a positive correlation existed between ball possession and the match result for England (CCF=0.92), France (CCF=0.37) and Italy (CCF=0.33).

There were positive autocorrelations in relation to the total passes for Spain (ACF=0.48) and Italy (ACF=0.07). The results of cross-correlation also showed that there were positive correlations between the total passes and the result for England (CCF=0.56) and Spain (CCF=0.22).

There were positive autocorrelations in relation to pass accuracy for England (ACF=0.04). The results of the cross-correlation also showed a positive correlation between pass accuracy and the match result for Germany (CCF=0.82), Spain (CCF=0.75) and England (CCF=0.49).

Figure 3 shows the autocorrelation and cross-correlation functions of the defensive-related indicators for the different teams.

There were positive autocorrelations in relation to aerial duels won for Spain (ACF=0.19) and France (ACF=0.15). The results of cross-correlation also showed that there were positive correlations in relation to aerial duels won and the result for Portugal (CCF=0.68).

There were also positive autocorrelations in relation to tackles won by England (ACF=0.25) and Portugal (ACF=0.03). The results of the cross-correlation also showed that there was a positive correlation between tackle won and the result for France (CCF=0.69).

## Discussion

The aims of this study were to investigate the consistency of performance for soccer teams which qualified for the quarter final stage of EURO 2012 and to analyze the association between performance indicators with the results of each match. The results in Table 1 show the average values between different performance indicators as a standard for top level national teams in Europe. The highest percentage of shot accuracy was 51% and for ball possession it was equal to 65%. For pass accuracy the highest level achieved was 88%. The results of autocorrelation function showed that among the qualified soccer teams, Spain, the eventual tournament winners, showed better consistency in all the goal-related indicators including shots, shot on target, and shot accuracy. For offensive-related indicators, Spain showed better consistency in relation to total ball retention (possession), total passes and corners. Spain was also very consistent in defensive-indicators in relation to aerial duels won. Italy as their respective opponent in the final showed better consistency in the tournament in relation to shots and shots on target for the goal-related indicators. In offensive-related indicators, Italy was consistent for total passes, long passes, crosses and corners won. Italy was also consistent in defensive-related indicators in relation to interceptions and tackles. Portugal who reached the semi-final showed consistency in relation to shot accuracy from the goal-related indicators. In offensive-related indicators, Portugal was consistent in relation to ball retention (possession) and crosses. Portugal was also consistent in the tournament in relation to interception from the defensive-related indicators. France showed consistency in relation to shots on target and shot accuracy from the goal-related indicators. France was also consistent for long passes from the offensive-related indicators and in relation to defensive-related indicators for aerial duels won. England was not consistent with the goal-related indicators, but showed consistency for the offensive-related indicators relating to long passes, pass accuracy and crosses and in relation to tackles won from the defensive-related indicators. Germany only showed consistency in relation to clearance from the defensive-related indicators. The remaining teams, Greece and the Czech Republic did not show consistency in any performance indicator.

Of all the teams, both Spain and Italy who reached the tournament final showed better consistency when compared to other teams especially in relation to the goal-related and offensive-related indicators. These findings somewhat support previous studies in relation to being successful with goal-related and offensive-related indicators and the respective successful performance in an international tournament soccer, such as the UEFA Euro tournaments. Indicators such as the total number of shots on goal, ball possession, the number of total shots and the total shots on target (Hughes and Franks, 2005; Castellano et al., 2012; Hook and Hughes, 2001) all appear critical in international soccer in Europe. However, it is impossible to assume that the same indicators will be of greatest significance in other international soccer tournaments (e.g. FIFA World Cup, African Cup of Nations). Tactically, there could be differences in these tournaments, possibly due to both different deployment of match tactics and differences in the ability of teams in these and other tournaments.

Specifically in relation to match results, the cross-correlation function showed that there were higher goal-related indicator associations in the match between Spain and France. In specific match results there were positive correlations in matches and offensive related indicators for France (in relation to possession, corners, long passes and crosses); England (in relation to corners, long passes and crosses), Portugal (in relation to the total passes, pass accuracy and long passes), Greece (in relation to long passes, cross accuracy and corners won), the Czech Republic (in relation to long passes) and Spain (in relation to pass accuracy and cross accuracy). Intuitively, pass accuracy and success would appear to be a tactical approach, based on a technical ability that led to Spain being successful in Euro 2008, the FIFA World Cup 2010 and Euro 2012. But this kind of a conclusion and interpretation requires an analysis of different tournaments to find a consistent manner for winning major soccer cups by a particular team like Spain. In relation to the defensive-related indicators and match success, France demonstrated consistency for duels won, England in relation to interception, tackles won and clearance, Greece in relation to duels won, interception, tackles made and clearance and Portugal in relation to interceptions, tackles and tackles won. These findings showed that each team had different playing patterns for successful performance in different matches, but lacked consistency in the tournament overall, with the exception of Spain. Results of our study also support the Luhtanen et al.’s (2001) analysis of EURO 2000 where the authors reviewed the relative strengths and weaknesses of all teams across different performance indicators. In their study, Luhtanen et al. (2001) demonstrated that France, the winner of the tournament, had the highest rating for passes received and for tackling, Italy’s main strengths were their defensive skills related to interceptions and tackling. The Netherlands had the best record with regard to ball retention (possession), passing and shooting. Germany performed well taking into account ball possession, passing and the number of goal scoring opportunities. The current analytical study showed that the winner of two successive Euro cups 2008 and 2012, Spain, still relied more on goal-related and offensive-related indicators to achieve the success such as shot, shot accuracy, pass accuracy and cross accuracy.

Based on findings of time series analysis it is demonstrated that both Spain and Italy used their consistent performance in successive matches in order to achieve the better results. Furthermore, it is useful to assess how the autocorrelation and cross-correlation functions showed the direction and degree of association of the performance indicators for the different teams. Analysis of Figures 1 to 3 showed that Spain demonstrated consistency and a positive correlation in the match result in relation to the total number of shots, as well as shots on target, shot accuracy, corners won and total pass completion. Other teams that were also successful in the tournament showed positive results in other areas: Italy in relation to corners, Portugal in relation to interception and France in relation to shots on target and long pass completion. It seems that regardless of the magnitude of association, Spain demonstrated regular and consistent performance in different indicators to be successful. Yet, their ultimate success came from excelling in goal-related indicators in order to achieve the best result in successive matches.

This study supported previous findings in different ways (Lago-Penas et al., 2010; Erkmen, 2009; Kannekens et al., 2011; Lago-Ballesteros and Lago-Penas, 2010; Tenga et al., 2009). Instead of comparative studies through means difference or discriminative analysis, we applied a time series analysis in order to code and examine the correlations of performance indicators throughout the tournament. These findings showed that goal-related and offensive-related indicators played a significant role in successful performance in international tournament soccer. However, one cannot state with any degree of accuracy that this is truly indicative of every international tournament. It would be useful to apply the same approach over multiple tournaments to assess if the successful indicators do change between tournaments. Intuitively, one may hypothesize that different indicators would have been more prominent when Greece won Euro 2004. Their style of play appeared to be somewhat different, but were the successful performance indicators different? Equally, one would not be accurately able to hypothesize that the successful performance indicators in top level European club soccer (e.g. The Premier League, La Liga, Bundesliga, Serie A) as well as the UEFA Champions League and UEFA Europa League would be the same as in international tournament soccer. Indeed, one is more likely to hypothesize that the successful indicators may be different in these competitions. Clubs are not restricted by geographical ‘talent pools’ as they can source players from anywhere globally and therefore, they may develop and deploy different systems in order to succeed.

The findings of the present study showed that Spain and Italy demonstrated greater performance consistency relative to other teams in half of the key performance indicators and that Spain’s performance consistency in some indicators played a higher role in their successful match and tournament result when compared to other teams.

The findings of this study have practical implications for coaches who work with players of different levels. The findings showed that relying on consistent tactics by top ranked soccer teams is an effective strategy applied in order to increase the chance for achieving the successful results.

## Figures and Tables

**Table 1:** Number of matches, mean and standard deviation of different key performance indicators in EURO 2012 (Bold show the first and second ranked teams in each indicator)

Performance Indicators	Spain	Italy	Germany	Portugal	France	England	Greece	Czech Republic
Shot	15.83 (6.55) 6	**16.83 (7.65)** 6	13.5 (4.35) 5	15.5 (5.91) 5	**16.25 (8.8)** 4	10 (4.54) 4	7.33 (1.15) 4	12.33 (3.51) 4
Shot on target	**6.66 (4.63)** 6	**5.33 (1.21)** 6	5 (2.16) 5	4.25 (3.5) 5	5.5 (3.87) 4	2.75 (2.36) 4	1.66 (.57) 4	4.33 (1.15) 4
Shot accuracy (%)	**51.26 (19.4)** 6	45.43 (10.86) 6	45.72 (12.5) 5	33.87 (23.61) 5	**47.2 (27.23)** 4	34.77 (13.23) 4	30.53 (4.79) 4	43.86 (10.17) 4
Possession (%)	**65.28 (7.47)** 6	51.33 (13.15) 6	**54.9 (5.62)** 5	39.85 (2.53) 5	54 (10) 4	40.65 (7.19) 4	43.33 (11.43) 4	51.53 (6.2) 4
Duel won (%)	**55.93 (5.92)** 6	48.55 (7.65) 6	**56.7 (6.3)** 5	48.8 (9.45) 5	45.85 (4.84) 4	51.55 (3.86) 4	52.55 (1.92) 4	47.13 (2.85) 4
Aerial duel won (%)	**58.83 (22.5)** 6	43.18 (21.22) 6	**57.27 (11.44)** 5	46.5 (22.4) 5	51.25 (7.6) 4	47.6 (7.46) 4	53.96 (2.72) 4	57.13 (8.28) 4
Interception	12.33 (3.01) 6	**23.5 (10.15)** 6	15.25 (2.75) 5	17.5 (10.9) 5	14.75 (4.57) 4	17.75 (3.2) 4	**21.66 (5.68)** 4	7 (1.73) 4
Corner	**6.66 (3.2)** 6	4.83 (4.21) 6	**6.25 (5.43)** 5	7.5 (2.38) 5	7 (3.36) 4	4 (1.41) 4	3 (2) 4	5 (1.52) 4
Total pass	**676.6 (112)** 6	461.5 (75) 6	**517.5 (80)** 5	324.75 (17) 5	511 (102) 4	353.5 (73) 4	323 (84) 4	420.3 (50) 4
Long pass (%)	7.06 (1.87) 6	10.91 (1.58) 6	8.62 (3.23) 5	**14.35 (2.17)** 5	9.22 (2.2) 4	11.62 (1.96) 4	**16.5 (1.21)** 4	12.16 (3.59) 4
Pass accuracy (%)	**88.46 (2.32)** 6	82.35 (3.24) 6	85.75 (3.04) 5	76.42 (2.76) 5	**86.87 (3.04)** 4	80.40 (4.45) 4	76.5 (7.06) 4	80.43 (2) 4
Cross	13.66 (4.27) 6	15.16 (8.77) 6	**24.5 (15.15)** 5	**22.5 (4.43)** 5	19.25 (5.85) 4	17.75 (2.06) 4	18.33 (4.16) 4	16 (5.56) 4
Cross accuracy (%)	23.45 (9.09) 6	**30.93 (8.95)** 6	23.5 (4.74) 5	15.95 (9.13) 5	25.25 (15.07) 4	**25.57 (9.8)** 4	14.93 (12.56) 4	23.26 (21.11) 4
Tackle	19.33 (2.94) 6	15.5 (3.39) 6	18.75 (8.61) 5	14 (2.94) 5	16.5 (3.41) 4	**20.5 (5.74)** 4	16.33 (2.08) 4	13.66 (6.8) 4
Tackle won (%)	**79.48 (10)** 6	73.4 (13.57) 6	76.42 (9.47) 5	70.4 (12.8) 5	77.4 (10.85) 4	73.2 (2.07) 4	**80.1 (7.03)** 4	77.66 (8.08) 4
Clearance	16.33 (2.87) 6	22.33 (10.81) 6	15.5 (9.46) 5	16.75 (7.32) 5	18.75 (4.5) 4	**24.25 (2.5)** 4	**29.33 (4.72)** 4	19 (10.14) 4

## References

[b1-jhk-38-213] Armatas V, Yiannakos A, Sileloglou P (2007). Relationship between time and goal scoring in soccer games: Analysis of three world cups. Int J Perform Anal Sport.

[b2-jhk-38-213] Carling C, Williams MA, Reilly T (2005). Handbook of soccer match analysis: A systematic approach to improving performance.

[b3-jhk-38-213] Castellano J, Casamichana D, Lago-Penas C (2012). The use of match statistics that discriminate between successful and unsuccessful soccer teams. Journal of Human Kinetics.

[b4-jhk-38-213] Dellal A, Chamari K, Wong DP, Ahmaidi S, Keller D, Barros R, Bisciotti GN, Carling C (2011). Comparison of physical and technical performance in European soccer match-play: FA Premier League and La Liga. Eur J Sport Sci.

[b5-jhk-38-213] Erkmen N (2009). Evaluating the heading in professional soccer players by playing position. J Strength Cond.

[b6-jhk-38-213] Hook C, Hughes M, CPA (Centre for Performance Analysis) (2011). Patterns of play leading to shots in Euro 2000. Pass.com.

[b7-jhk-38-213] Hughes M, Bartlett R (2002). The use of performance indicators in performance analysis. J Sport Sci.

[b8-jhk-38-213] Hughes M, Bartlett R, Hughes M, Frank IM (2008). What is performance analysis. The essentials of performance analysis: An introduction.

[b9-jhk-38-213] Hughes M, Franks IM (2005). Analysis of passing sequences, shots and goals in soccer. J Sports Sci.

[b10-jhk-38-213] Kannekens R, Elferinks-Gemser T, Visscher C (2011). Positioning and deciding: Key factors for talent development in soccer. Scand J Med Sci Spor.

[b11-jhk-38-213] Lago-Ballesteros J, Lago-Penas C (2010). Performance in team sports: Identifying the keys to success in soccer. Journal of Human Kinetics.

[b12-jhk-38-213] Lago-Penas C, Lago-Ballesteros J, Dellal A, Gomez M (2010). Game-related statistics that discriminated winning, drawing and losing teams from the Spanish soccer league. J Sport Sci Med.

[b13-jhk-38-213] Lago-Penas C, Lago-Ballesteros J, Rey E (2011). Differences in performance indicators between winning and losing teams in the UEFA champions league. Journal of Human Kinetics.

[b14-jhk-38-213] Liebermann DG, Franks IM, Hughes M, Frank IM (2008). Video feedback and information technologies. The essentials of performance analysis: An introduction.

[b15-jhk-38-213] Luhtanen P, Belinskij A, Hayrinen M, Vanttinen T (2001). A comparative tournament analysis between the Euro 1996 and 2000 in soccer. Int J Perform Anal Sport.

[b16-jhk-38-213] Prozone. Analysis: Spain 2006-12 - The Evolution of Success. The Prozone Newsletter; 2012

[b17-jhk-38-213] Rampinini E, Couts AJ, Castagna C, Sassi R, Impellizzeri FM (2007). Variation in top level soccer match performance. Int J Sports Med.

[b18-jhk-38-213] Rampinini E, Impellizzeri FM, Castagna C, Coutts AJ, Wisloff U (2009). Technical performance during soccer matches of the Italian series A league: Effect of fatigue and competitive level. J Sci Med Sport.

[b19-jhk-38-213] Scoulding A, James N, Taylor J (2004). Passing in the soccer world cup 2002. Int J Perform Anal Sport.

[b20-jhk-38-213] Stergiou N (2004). Innovative analyses of human movement.

[b21-jhk-38-213] Shafizadeh M, Shirley G, Sproule J, McMorris T (2012). An exploratory analysis of losing possession in professional soccer. Int J Perform Anal Sport.

[b22-jhk-38-213] Stanhope J (2001). An investigation into possession with respect to time, in the soccer world cup 1994. Notational analysis of sport III.

[b23-jhk-38-213] Taylor JB, Mellalieu SD, James N, Shearer DA (2008). The influence of match location, quality of opposition, and match status on technical performance in professional association football. J Sport Sci.

[b24-jhk-38-213] Tenga A, Kanstad D, Ronglan LT, Bahr R (2009). Developing a new method for team match performance analysis in professional soccer and testing its reliability. Int J Perform Anal Sport.

[b25-jhk-38-213] Yue Z, Broich H, Seifriz F, Mester J (2008). Mathematical analysis of a soccer game, Part I: Individual and collective behaviors. Stud Appl Math.

[b26-jhk-38-213] Nuviala A, Gómez-López M, Pérez JA, Nuviala R (2011). Lifestyle and Physical Education. J Human Kinetics.

[b27-jhk-38-213] Olympiou A, Jowett S, Duda J (2008). The psychological interface between the Coach - created motivational climate and the Coach – Athlete Relationship in Team Sport. Sport Psychol.

[b28-jhk-38-213] Papaioannou AG, Ampatzoglou G, Kalogiannis P, Sagovits A (2008). Social agents, achievement goals, satisfaction and academic achievement in youth sport. Psychol Sport Exercise.

[b29-jhk-38-213] Pelletier LG, Fortier MS, Vallerand RJ, Tuson KM, Brière NM, Blais MR (1995). Toward a new measure of intrinsic motivation, extrinsic motivation, and amotivation in sports: The Sport Motivation Scale (SMS). J Sport Exercise Psy.

[b30-jhk-38-213] Roberts GC, Roberts GC (2001). Understanding the dynamics of motivation in physical activity: The influence of achievement goals and motivational processes. Advances in motivation in sport and exercise.

[b31-jhk-38-213] Roberts GC, Treasure DC, Balagué G (1998). Achievement goals in sport: The development and validation of the Perception of Success Questionnaire. J Sport Sci.

[b32-jhk-38-213] Sallis JF, McKenzie TL, Kolody B, Lewis M, Marshall S, Rosengard P (1999). Effects of health-related physical education on academic achievement: Project SPARK. Res Q Exercise Sport.

[b33-jhk-38-213] Scanlan TK, Lewthwaite R (1986). Social psychological aspects of competition for male youth sport participants: IV. Predictors of performance outcomes. J Sport Psychol.

[b34-jhk-38-213] Scanlan TK, Simons JP, Carpenter PJ, Schmidt GW, Keeler B (1993). The sport commitment model: Measurement development for the youth-sport domain. J Sport Exercise Psy.

[b35-jhk-38-213] Smith AL, Balaguer I, Duda JL (2006). Goal orientation profile differences on perceived motivational climate, perceived peer relationships, and motivation-related responses of youth athletes. J Sports Sci.

[b36-jhk-38-213] Sousa C, Torregrosa M, Viladrich C, Villamarín F, Cruz J (2007). The commitment of young soccer players. Psicothema.

[b37-jhk-38-213] Torregrosa M, Cruz J, Sousa C, Viladrich C, Villamarín F, Garcia Mas A, Palou P (2007). Fathers and mothers′ influence on sport commitment in youth footballers. Rev Lat Am Psicol.

[b38-jhk-38-213] Weiss MR, Ferrer-Caja E, Horn TS (2002). Motivational orientations in sport. Advances in Sport Psychology.

[b39-jhk-38-213] Wuerth S, Lee M, Alfermann D (2004). Parental Involvement and athletes’ career in youth Sport. Psychol Sport Exercise.

